# Insulinoma Presenting as Seizures: Challenges of Managing a Rare Disease in a Resource-challenged Setting

**DOI:** 10.1210/jcemcr/luad162

**Published:** 2023-12-19

**Authors:** David O Soyoye, Segun A Atolani, Tajudin A Adetunji, Olusegun I Alatise

**Affiliations:** Department of Medicine, Obafemi Awolowo University, Ile-Ife, Osun 220282, Nigeria; Department of Medicine, Obafemi Awolowo University Teaching Hospital, Ile-Ife 220213, Nigeria; Department of Medicine, Obafemi Awolowo University Teaching Hospital, Ile-Ife 220213, Nigeria; Department of Medicine, Obafemi Awolowo University, Ile-Ife, Osun 220282, Nigeria; Department of Medicine, Obafemi Awolowo University Teaching Hospital, Ile-Ife 220213, Nigeria; Department of Surgery, Obafemi Awolowo University, Ile-Ife, Osun 220282, Nigeria; Department of Surgery, Obafemi Awolowo University Teaching Hospital, Ile-Ife 220213, Nigeria

**Keywords:** insulinomas, neuroendocrine tumors, seizures, hypoglycemia, obesity

## Abstract

Insulinomas are functioning pancreatic neuroendocrine tumors (NETs). They secrete insulin, and hence, present with hypoglycemia. We report a case of insulinoma in a 16-year-old girl presenting as seizures. She was initially managed at a private clinic and later commenced on carbamazepine when convulsion persisted. Convulsions were generalized, associated with dizziness and altered sensorium, often preceded by hunger and physical exertion, but relieved by the intake of carbonated drinks and fruit juice. She was referred to the neurology clinic when seizures persisted, despite the use of anticonvulsant. She was later referred to the endocrine clinic on suspicion of insulinoma when her random blood glucose (BG) was found to be low during an episode of convulsion. She was moderately obese but other examination findings were normal. She had a 72-hour prolonged fast, which was terminated when hypoglycemia (BG = 2.2 mmol/L) ensued after 12 hours, with elevated serum insulin and C-peptide. Abdominal magnetic resonance imaging scan showed a pancreatic tumor suggestive of insulinoma. She subsequently had distal pancreatectomy performed with complete resolution of symptoms. Unusual presentation of insulinoma may delay diagnosis, resulting in wastage of resources with increased morbidities and mortality.

## Introduction

Insulinomas are insulin-secreting neuroendocrine tumors (NETs) of the pancreas, with attendant hypoglycemic effects. Although they are rare, they are the most common pancreatic endocrine tumors [1, 2]. Most cases of insulinoma occur sporadically; it can, however, occur as one of the components of multiple endocrine neoplasia (MEN). A 90% rule has been reported in insulinoma stating that 90% of the cases are sporadic, 90% are solitary, 90% are intrapancreatic, and 90% are benign [1, 2].

Insulinoma can occur at any age, with a mean age of 47 years, and a slight female preponderance (1.4:1) [1, 2]. Prevalence is estimated to be about 3 to 4 per million people in the general population. There is a paucity of data about insulinoma in sub-Saharan Africa, especially Nigeria. A cursory literature search showed that only one case of insulinoma has ever been reported in a Nigerian; by Akanji et al [3], in a pregnant woman in the first trimester.

Patients with insulinoma usually present with autonomic and neuroglycopenic symptoms of hypoglycemia, which include diaphoresis, tremor, palpitations, confusion, behavioral changes, visual disturbances, and seizure. Symptoms typically occur several hours after a meal, usually at night or early in the morning. Symptoms may also be precipitated by physical exertion and alcohol intake. Another important presentation of insulinoma is weight gain [1]. Insulinoma should be suspected in patients who fulfil the classic Whipple triad: classic symptoms of hypoglycemia, documented biochemical evidence of hypoglycemia with plasma glucose of less than 50 mg/dL (2.7 mmol/L), and prompt resolution of symptoms following administration of glucose [1].

## Case Presentation

The patient is a 16-year-old secondary school female student who was referred to the endocrinology clinic on account of recurrent convulsions and excessive weight gain of 18 months’ duration.

The first episode of seizures occurred in school and was said to have been heralded by a prodrome of palpitations and dizziness. Seizures abated after few minutes on intravenous fluid (name unknown) in a private clinic, with marked improvement in clinical status.

Subsequent episodes of seizures typically lasted for a few minutes, were generalized tonic-clonic in nature, were preceded either by hunger or physical exertion, and associated with tremors, palpitations, postictal sleep, and muscle aches. Symptoms were largely preventable by regular consumption of sugar-containing carbonated drinks. There was no diurnal variation in the symptoms, and there was no preceding history of head injury or delay in the attainment of developmental milestones. The patient also has no known family history of seizure disorder.

There has been progressive weight gain during this period, with recurrent bouts of hunger pangs and hyperphagia. There was no history suggestive of depression or suicidal intent.

She was initially managed at a peripheral hospital for seizure disorder and was placed on carbamazepine. She was then referred to the neurology unit of our hospital for expert management on a tentative diagnosis of hypothyroidism from a clinic she later presented due to increasing episodes of seizures and low blood glucose (BG) readings.

During evaluation at the neurology clinic, the patient had sweaty spells and palpitations with a random BG of 2.7 mmol/L(48.6 mg/dL). Symptoms promptly resolved with ingestion of a sugar-containing carbonated drink, and she was referred to the endocrinology clinic on suspicion of insulinoma.

Examination findings at the endocrinology clinic were that of a young woman with a body mass index of 37.1 (body weight 101.1 kg, height 165 cm). There were no striae or easy bruising. Other examination findings were essentially normal.

## Diagnostic Assessment

Biochemical assessment was initially delayed for about 1.5 years due to financial constraints and the patient’s father's health challenges. She was admitted for 72-hour prolonged fast, which was terminated after 12 hours when she developed symptoms of hypoglycemia with BG of 2.2 mmol/L(39.6 mg/dL). At termination of fast, serum C-peptide was elevated at 5.85 ng/mL (1.95 nmol/L) (normal reference: 0.5-2.7 ng/mL [0.2-0.9 nmol/L]), and serum insulin was 52.5 µU/mL(364.6 pmol/L) (normal reference: 2-15 µU/mL [13.9-104.2 pmol/L]). Other biochemical tests, including serum calcium, thyroid hormones, gonadotrophin levels, and renal and liver function tests, were within normal.

**Figure 1.**
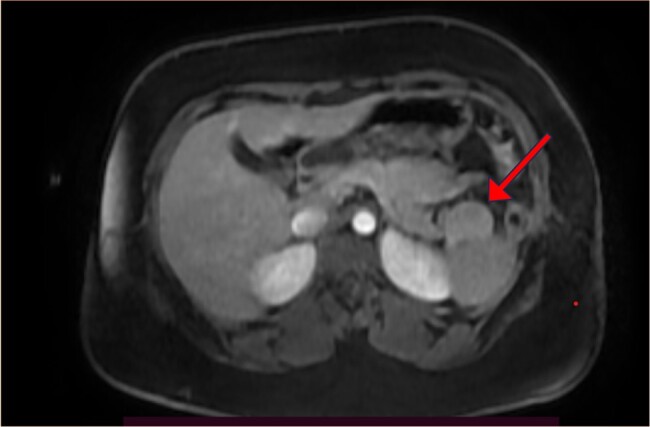
Axial section of the abdominal magnetic resonance imaging scan—an oval-shaped, well-marginated, T1-isointense to splenic parenchymal, T2-hyperintense mass lesion with no restricted diffusion in diffusion-weighted imaging/apparent diffusion coefficient images, measuring 3.64 × 3.72 × 2.63 cm in H × T × AP diameter.

Abdominopelvic ultrasound scan showed normal findings. Abdominal magnetic resonance imaging scan (MRI) (Fig. 1) showed a tumor at the tail of the pancreas suggestive of insulinoma; other abdominal structures were normal.

## Treatment

**Figure 2.**
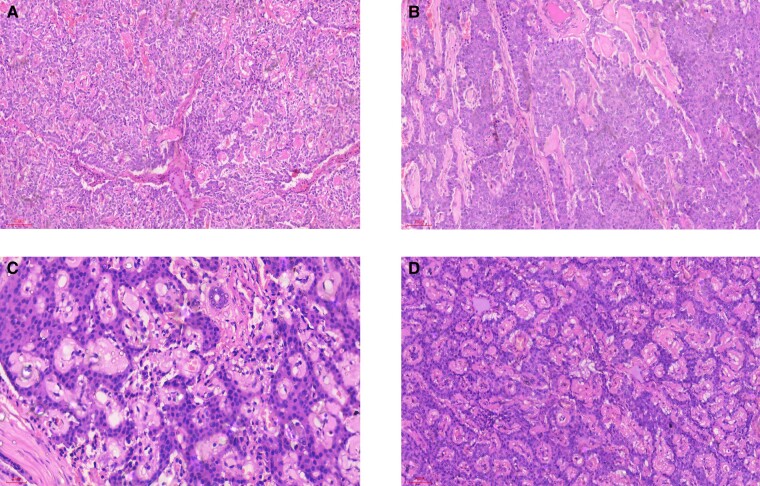
A to D, Histopathology of the resected pancreatic tissue. Microscopic sections show a fairly circumscribed and encapsulated lesion composed of trabecules, cords, and solid sheets of monotonous cuboidal cells with moderate cytoplasm and centrally located nuclei with dispersed chromatin pattern, giving it a “salt and pepper” appearance. These cells are all disposed in a background of acellular faintly eosinophilic stroma (? amyloid). There are no areas of atypia, necrosis, or invasion.

The patient was counseled on the need to take small, starchy carbohydrates frequently, to monitor her BG using a glucometer, and to avoid strenuous exercises before referral to the general surgeons. While awaiting surgery, she was placed on phenytoin (due to nonavailability of diazoxide and octreotide in Nigeria). This was discontinued due to anorexia, which resulted in symptoms of hypoglycemia. She subsequently had an open distal pancreatectomy performed with complete resolution of symptoms. Histopathologic examination showed a NET, most probably insulinoma (Fig. 2).

## Outcome and Follow-up

She initially developed hyperglycemia postoperatively, necessitating the use of insulin for a few weeks. She continued to monitor her BG using the glucometer, and has been euglycemic for almost 8 months after the operation. She was also counseled on the need for regular physical exercise and calorie reduction, for weight management.

## Discussion

In this report, we present a rare case of insulinoma in a teenage girl misdiagnosed as seizure disorder in a peripheral hospital. We also highlight some challenges encountered while managing this neuroendocrine tumor. To the best of our knowledge, this is the first case of a successful management outcome of insulinoma in a Nigerian, after a case was initially reported 30 years ago by Akanji et al [3] in a 30-year-old pregnant woman that proved fatal.

Given the refractory nature of the seizure in our patient, coupled with recurrent symptoms of hypoglycemia and demonstration of the Whipple triad, a tentative diagnosis of insulinoma was entertained. This was further reinforced by a history of unintended weight gain. Basic investigations were normal, including serum electrolytes, calcium and phosphate, etc.

Although the definitive 72-hour prolonged fast was started in this patient, it was terminated at about the 12th hour due to development of symptoms of hypoglycemia, and a capillary BG result of 2.2 mmol/L (39.6 mg/dL). The diagnosis of insulinoma was further strengthened by a demonstration of elevated insulin and C-peptide during the hypoglycemia. A proinsulin assay was not performed because of cost. A 72-hour fast, which tests the integrity of the patient's endogenous suppression of insulin in the setting of hypoglycemia, is the gold standard for diagnosis [4]. Only very few patients are able to tolerate the fasting for 72 hours, with most patients developing symptoms of hypoglycemia within 24 hours of initiating the test, thus, leading to termination of the procedure. Failure of appropriate insulin suppression in the presence of hypoglycemia substantiates an autonomously secreting insulinoma, especially when the use of sulfonylureas has been excluded [2].

The biochemical diagnosis of endogenous hyperinsulinemia can be made when the following parameters are found [5]:

- Serum insulin levels greater than or equal to 3.0 µU/mL (18 pmol/L);
- Glucose levels less than or equal to 55 mg/dL (3.0 mmol/L);
- C-peptide levels greater than or equal to 0.6 ng/mL (0.2 nmol/L);
- Proinsulin levels greater than or equal to 5 pmol/L (0.05 ng/mL); and
- β-hydroxybutyrate levels less than or equal to 2.7 mmol/L (28 mg/dL)

To assist with tumor localization and guide surgical therapy, an abdominal MRI scan was conducted. MRI is one of the imaging modalities of choice in NETs, given its noninvasive nature and better sensitivity [6]. In a study by Zhu et al [7], MRI was shown to demonstrate higher tumor conspicuity and was superior to the computed tomography (CT) in depicting the tumor-to-duct distance.

Localization of the tumor is an important aspect of the evaluation of patients with insulinoma, and various imaging modalities have been described, including noninvasive procedures such as abdominal ultrasound, CT, and MRI of the abdomen. Although abdominal ultrasound is readily available, its sensitivity is relatively low when compared with CT scan or MRI [6]. Radiolabeling with glucagon-like peptide 1 (GLP-1) analogues (eg, ^111^In-DOTA-exendin-4) has also been found to be helpful in tumor localization; however, this is not useful in malignant insulinomas, as these tumors do not express GLP-1 receptors [6]. Invasive procedures such as the endoscopic ultrasound scan and arterial stimulation with venous sampling (ASVS) are more accurate and superior to the noninvasive procedures in tumor localization [2]. Most of these investigations (including CT and MRI scan) are not readily available in most centers in our settings. This may result in delayed diagnosis and management.

Our patient was unable to take either diazoxide or octreotide, which were prescribed during the pendency of surgery due to scarcity of the drugs and paucity of funds. Phenytoin was used as an alternative because of its availability and cheaper cost. However, it was discontinued due to nausea. Phenytoin reduces the secretion of insulin, hence, its utility in insulinoma [6, 8]. Verapamil and glucocorticoids are other alternatives in drug management of insulinoma [6]. Though these are available in our environment, they were not considered for this patient because of their side effect profile (especially the propensity to cause weight gain), as our patient had a body mass index in the moderate obesity range.

Open pancreatectomy was performed after a detailed preoperative assessment. While laparoscopic technique remains favored because of fewer complications and cosmetic appearance, radical resection may be required for large tumors, multiple lesions, suspected malignant tumors, or when the tumor is near the main pancreatic duct [2, 9]. Although the tumor was not particularly large in this case, local experience favored the choice of an open procedure.

Surgery offers the best curative option in the management of insulinomas, and it is the most commonly employed modality of management [2, 9]. Occurrence of hyperglycemia after resection of insulinomas is common, and may be due to the downregulation of glucose transporter 2 and GLP1 receptor in the nontumor islet cells [10, 11].

Medically, therapy with drugs like diazoxide, octreotide, and phenytoin are frequently used for preoperative correction of hypoglycemia, or in malignant insulinomas that are not amenable to surgery [1, 2]. The use of arterial embolization, radiofrequency ablation, cryoablation, high-intensity focused ultrasound, and irreversible electroporation in the management of nonresectable insulinomas has also gained some popularity in the last few decades, but these are largely unavailable in Nigeria [6].

This case highlights some challenges in managing rare disorders such as insulinoma in resource-challenged environments, which prevail in most countries in sub-Saharan Africa. Nonavailability of required investigations and treatments are some of the challenges identified. This is coupled with poor coverage by the national health insurance programs, which results in direct payments by patients, with resultant poor health outcomes.

Delayed diagnosis and financial constraint are key contributors to the delay in the treatment of this patient. Although insulinoma is rare, it can be fatal if undiagnosed. It is imperative for medical practitioners to consider hypoglycemia and its causes, especially rare causes such as insulinoma, in the evaluation of patients with seizures.

Our case demonstrates the need for a high index of suspicion for insulinoma through meticulous evaluation of patients, and an appropriate and timely intervention. There is a need to continue to train medical personnel attending to patients at primary and secondary health care centers on the need to promptly refer difficult and atypical cases to improve survival, as demonstrated in this case.

## Learning Points

- Insulinoma is a rare pancreatic neuroendocrine tumor that usually presents with features of hypoglycemia.
- A high index of suspicion is needed for early diagnosis, especially when it presents mainly with an uncommon feature, such as seizures.
- The 72-hour prolonged fast with measurement of blood glucose, serum insulin, C-peptide, and proinsulin levels is a definitive diagnostic test for insulinomas, but may need to be terminated earlier if hypoglycemia sets in, and modified on account of cost and availability of analytes.
- Laparoscopic resection of the tumor is now favored on account of its fewer complications and cosmetic appearance; however, open surgery may still be considered when expertise for laparoscopic surgery is not available, or when the tumor is large, suspiciously malignant, multiple, or near the main pancreatic duct.

## Data Availability

Data sharing is not applicable to this article as no data sets were generated or analyzed during the current study.

## Abbreviations

BG: blood glucose
CT: computed tomography
GLP-1: glucagon-like peptide 1
MRI: magnetic resonance imaging
NET: neuroendocrine tumor
